# Imaging with a small number of photons

**DOI:** 10.1038/ncomms6913

**Published:** 2015-01-05

**Authors:** Peter A. Morris, Reuben S. Aspden, Jessica E. C. Bell, Robert W. Boyd, Miles J. Padgett

**Affiliations:** 1 School of Physics and Astronomy, University of Glasgow, University Avenue, Kelvin Building, Glasgow G12 8QQ, UK; 2 Department of Physics, University of Ottawa, Ottawa, Ontario, Canada K1N 6N5; 3 The Institute of Optics and Department of Physics and Astronomy, University of Rochester, Rochester, New York 14627, USA

## Abstract

Low-light-level imaging techniques have application in many diverse fields, ranging from biological sciences to security. A high-quality digital camera based on a multi-megapixel array will typically record an image by collecting of order 10^5^ photons per pixel, but by how much could this photon flux be reduced? In this work we demonstrate a single-photon imaging system based on a time-gated intensified camera from which the image of an object can be inferred from very few detected photons. We show that a ghost-imaging configuration, where the image is obtained from photons that have never interacted with the object, is a useful approach for obtaining images with high signal-to-noise ratios. The use of heralded single photons ensures that the background counts can be virtually eliminated from the recorded images. By applying principles of image compression and associated image reconstruction, we obtain high-quality images of objects from raw data formed from an average of fewer than one detected photon per image pixel.

## Abstract (web summary)

Advances in low-light-level imaging techniques have shown that imaging in the one photon per pixel regime is possible. Here, Morris *et al*. demonstrate high-quality image reconstruction using ghost and heralded imaging with less than one photon per image pixel with a time-gated intensified camera.

## Graphical abstract

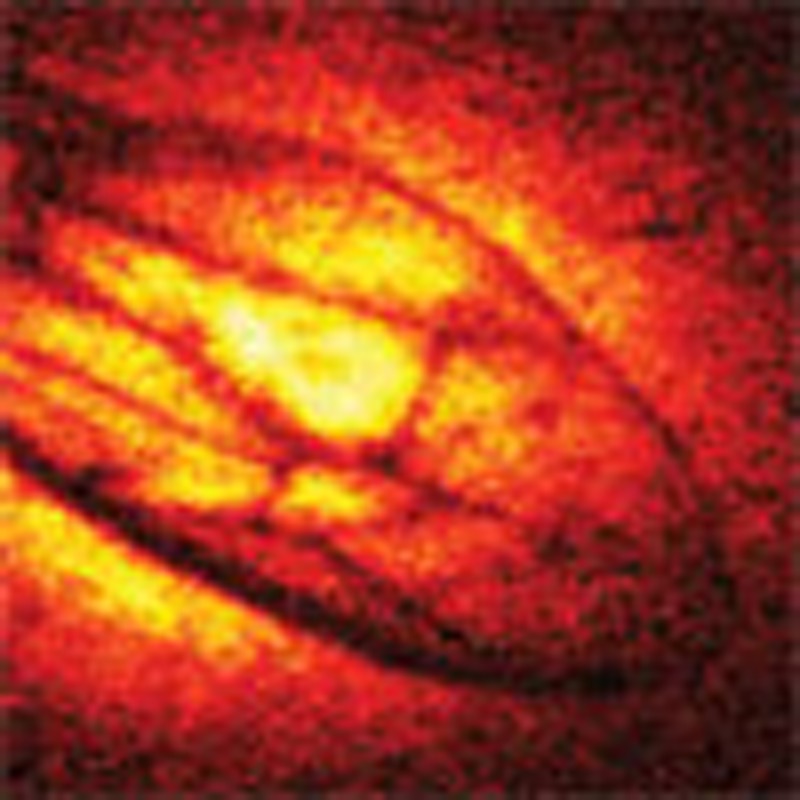

Imaging at very low-light-levels has applications spanning many diverse fields of interest including biological imaging and covert security protocols. A typical image taken with a conventional camera captures ~10^12^ photons^1^, but what is the minimum number of photons that it takes to form an image? Advances in imaging techniques invite a plausible imaging regime of one photon per pixel. It is this ultralow-photon flux regime that this paper investigates.

The photons generated through the spontaneous parametric downconversion (SPDC) process have served as an illumination source for many low-light-level applications^2^,^3^,^4^. The SPDC process provides an easily manipulated source of photon pairs with strong correlations in the spatial degrees of freedom of the photons^5^. Furthermore, the photons can be separated using a beam splitter (BS) into two different optical paths or arms of an experiment. These correlations have been exploited in several single-photon imaging experiments, including quantum ghost imaging (GI)^6^ and quantum interference imaging^7^. One method for utilizing these correlations is an imaging system where the detection of one of the photons in the generated photon pair is used to herald the arrival of its partner. In such systems, the heralding detector is a large area, single-pixel detector while the other, the imaging detector, is spatially resolving. One has two options for object placement: either place the object in the same arm of the experiment as the imaging detector as per a standard imaging system, or by exploiting the spatial correlations between the two photons, place the object in the heralding detector arm, as demonstrated by Pittman *et al*.^6^ in a display of quantum GI. Despite the use of a SPDC source, one should note that correlations within a single measurement basis (in this case the position basis) are not in themselves proof of entanglement but rather a utilization of entanglement^8^,^9^.

Traditionally, within a quantum GI system, the spatially resolving detector has been a scanning single-pixel detector. However, basing the system on a single scanning detector fundamentally limits the detection efficiency to 1/*N*, where *N* is the number of pixels in the image. Overcoming this limitation by using a detector array to increase the detection efficiency enables the acquisition of images while illuminating the sample with *N*-times fewer photons. This reduction in the required illumination flux is potentially beneficial for applications in biological imaging, where bleaching or sample damage can occur from a high photon flux, and also in security, where reducing the photon flux can make the system covert. Indeed, there are a number of recent papers using detector arrays with single-photon sensitivity^10^,^11^,^12^,^13^.

Our camera-enabled, time-gated imaging system uses the detection of one of the photons, the ‘heralding’ photon, from a downconverted photon pair by a single-pixel detector, to trigger the detection of the position-correlated photon by an intensified CCD camera (ICCD). We characterize this imaging system for two different system configurations, either with the object in the heralding arm, as per GI, or the object in the camera arm of the system. The heralding nature of our imaging system enables us to count the number of single photons present in each recorded image. To utilize the low-photon flux capabilities of our system, reconstruction techniques are applied to our data that allow us to obtain images using undersampled data sets consisting of an average of fewer than one photon per image pixel. We achieve this by operating within the constraints of Poissonian statistics and exploiting the sparsity of our images in the spatial frequency domain to subjectively improve the quality of the reconstructed images. With optimization, we are able to obtain images of our biological sample using fewer detected photons than there are pixels in the image. The combination of our imaging system and reconstruction techniques allow for the acquisition of images with very low-photon flux illumination. This minimization of photon exposure may have application to covert imaging applications or where the light itself can damage or otherwise modify the object.

## Results

### Experimental methods

**Figure 1:**
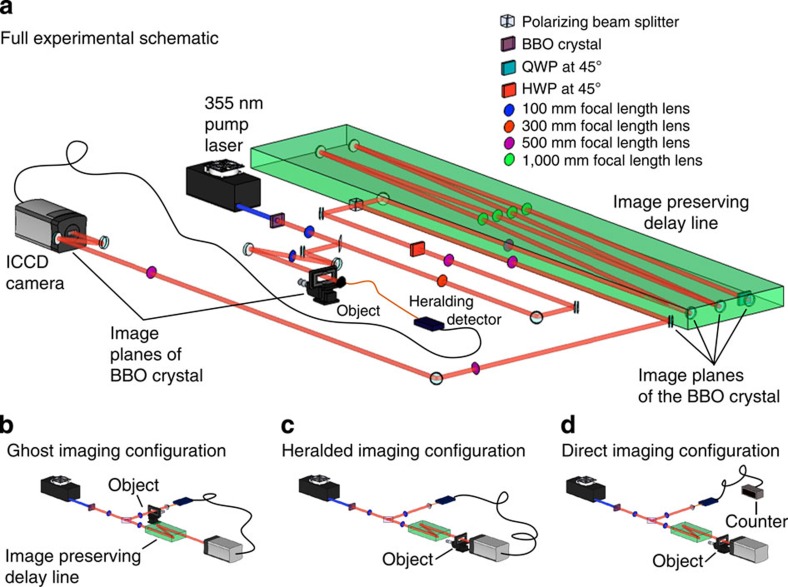
Experimental schematics. (**a**) Full schematic of our imaging system. A 355 nm laser pumps a *β*-barium borate crystal to produce collinear downconverted photon pairs at 710 nm. The output facet of the crystal is imaged onto the plane of the microscope slide (containing our object) and the ICCD camera. The image-preserving delay line is necessary to compensate for the electronic delays in the triggering mechanism. (**b**–**d**) Simplified schematics of each imaging configuration. (**b**) Ghost-imaging configuration: the object is placed in the heralding arm and the camera is triggered by each photon detection at the heralding detector. (**c**) Heralded imaging configuration: the object is placed in the camera arm and the camera is again triggered by each photon detection at the heralding detector. (**d**) Direct imaging configuration: the object is placed in the camera arm but the camera is triggered by an internal trigger mechanism, with the same trigger rate as the number of singles detected at the counter in the heralding arm.

Our imaging system is similar to that reported in refs 13, 14. We use correlated photons generated by SPDC and a multipixel ICCD triggered by a single-photon avalanche detector (SPAD), the latter acting as the heralding detector. The source of our downconverted photons is a 3-mm-long, non-linear *β*-barium borate crystal, cut for type-I phase matching and pumped by a horizontally polarized, quasi continuous-wave laser at 355 nm. The laser output is spatially filtered and recollimated to produce a ≈1.2 mm (full-width half-maximum) fundamental Gaussian beam at the input facet of the downconversion crystal. The generated near-collinear beam of frequency-degenerate, downconverted photons is selected through the use of high-transmission interference filters with a 10 nm bandwidth centred on 710 nm. Due to the large transverse Gaussian profile of the pump beam and short length of the downconversion crystal, our downconverted photons exhibit strong correlations over a wide range of spatial modes^15^. Our pairs of correlated photons are separated using a pellicle BS that directs the separated photons into the camera arm and the heralding arm of the system. Each arm has a magnification *M*=3 between the plane of the downconversion crystal and the planes of the object/camera. The object is placed on a microscope slide positioned in the image plane of the crystal in either the heralding or camera arm, depending on the desired system configuration (see Fig. 1). The camera is also positioned in an image plane of the crystal/object. Our object is thus illuminated by a spatially incoherent, multimode beam with a full-width half-maximum of ~3.6 mm. The photons in the heralding arm are collected by a detector consisting of an × 4 objective lens, a 400 μm core multimode fibre and a SPAD. This heralding detector registers the detection of a photon but records no spatial information.

There are two timing measures of relevance when using an ICCD camera. The first of these is the intensifier gate width, during which any single input photon is amplified by the intensifier and the event recorded on the CCD chip. This gate width has a typical duration of several nanoseconds. The second is the CCD exposure time, which is the time between each readout of the CCD chip, typically several seconds. Of course, the intensifier can fire many times during each exposure and thus each frame that is read out is an accumulation of all the detected single-photon events acquired during the exposure time.

The intensifier of the ICCD camera can be triggered using either an external pulse from the heralding SPAD or by using an internal pulse generator. When triggered using an external pulse, the gate width of the intensifier is set by the width of the input transistor–transistor logic pulse from the SPAD (≈15 ns). To ensure that the photons detected at the heralding detector and at the camera are from the same correlated photon pair, the electronic delay in the ICCD triggering mechanism must be compensated for by the introduction of additional optical path length in the camera arm^13^. In our system, we compensate for this electronic delay by introducing a 22 m image-preserving, free-space delay line. We attenuate the pump beam to all but eliminate the probability of generating multiple photon pairs per pump laser pulse, ensuring that we only record one photon per gating of the ICCD camera.

### Image acquisition

We acquire images using three different system configurations as shown in Fig. 1. In the GI configuration, the object is placed in the heralding arm, and the camera is triggered externally by the signal from the heralding detector. Thus an image of the object is formed on the camera, despite none of the imaged photons having interacted with the object. For the heralded imaging (HI) configuration, the camera is again triggered by the external trigger pulse, but the object is placed in an intermediate imaging plane in the camera arm. The camera is therefore triggered for each detected single photon yet the image consists only of the correlated photons that pass through the object. For comparison, we also show direct imaging (DI), where the camera is triggered using its internal trigger mechanism. In this last configuration the image consists only of the subset of photons that pass through the object and arrive at the camera during the camera trigger window by random chance. These three system configurations are illustrated in Fig. 1.

**Figure 2:**
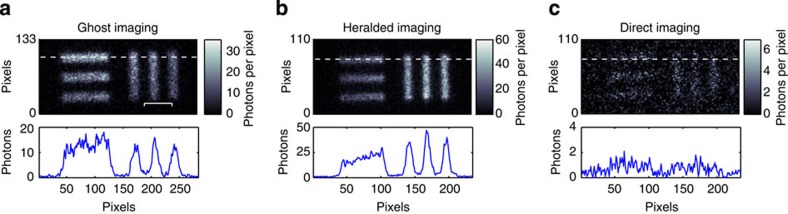
Acquired images using the different imaging configurations. (**a**) Ghost-imaging (GI) configuration with the object in the heralding arm and the camera triggered by the heralding detector. (**b**) Heralded imaging (HI) configuration where the object is in the camera arm and the camera is triggered by the heralding detector and (**c**) Direct imaging configuration, where the object is in the camera arm and the camera is internally triggered. It can be seen that we obtain a clear image with high contrast in both the GI and HI configurations, while the random nature of the detection mechanism in the direct imaging configuration yields only a very low contrast image. Scale bar, 650 μm.

The images shown in Fig. 2 are formed from the sum of 900 frames each of 2 s exposure, during which time the camera intensifier fires for every trigger pulse received, either from the heralding detector or the internal trigger mechanism. The CCD chip is air cooled to −30 °C, and we work with a region of interest of 600 × 600 pixels, covering an area of (7.8 × 7.8) mm^2^. The exposure time is chosen to ensure that each acquired frame is photon sparse, that is, ≪1 photon event per pixel^14^. Photon counting is possible by applying a binary threshold to the value of each pixel in the data read from the ICCD, a fuller description of which is provided in ref. 13. As part of this photon counting procedure, we calculate a noise probability per pixel by acquiring 100 triggered frames with the camera shutter closed. Plotting a histogram of the output signal from the camera allows us to set a threshold, a signal over which we define a photon. Using this threshold, we calculate a dark-count probability per pixel arising from the camera readout noise, which we calculate to be 5 × 10^−4^ per frame.

Figure 2 shows the images acquired using each of the system configurations. For both the GI and HI configurations, we obtain a clear image of the test target with an image contrast of ≈0.7, where we define the image contrast as


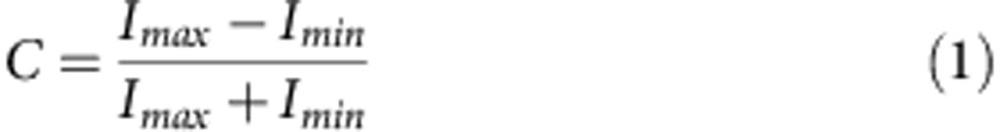


By comparison, when using the DI configuration, only a very faint image of the object is obtained with a contrast of ≈0.2. This reduced contrast for the DI configuration results from the repetition rate of the laser and the periodic nature of the intensifier trigger being entirely independent. Therefore, the arrival of the downconverted photon and the regular firing of the camera intensifier window only occasionally coincide, and thus the coincidence nature of the system is lost leading to a very low detection efficiency.

Closer inspection of the GI and HI images reveals a slight difference in scale, resulting from the magnification in the two arms not being quite the same. One also notes that although the total number of image photons is similar in the two cases, the GI configuration was obtained with fewer triggers of the intensifier than the HI configuration. This difference arises because although the photon pair generation rate in the two configurations is the same, when the partially transmitting object is placed in the heralding arm the trigger rate is reduced in proportion to the transmission of the object. For high flux rates, the GI configuration may therefore prove to be advantageous since it makes a lower technical demand on the ICCD camera.

### Optimization of reconstructed image

For the imaging system to be applied in ultra-low light conditions, one fundamental question is ‘how many photons does it take to form an image?’ Simplistically speaking, one requires many photons per pixel (typically 10,000 photons per pixel for a conventional imaging system), so that the intensity of each pixel is not unduly subject to the Poissonnian statistics associated with the quantization of the number of individual detected photon^16^. However, when an image is sparse in a chosen basis, it is possible to implement compressive techniques to store or even reconstruct the image from far fewer measurements than this simplistic statement implies^17^,^18^,^19^,^20^. These reconstruction techniques have also been shown to enhance efficiency in applications requiring the exploration of a large state space, for example, in quantum state tomography^21^ and more recently in quantum imaging. This latter use of compressive techniques in a quantum imaging system allowed an image to be reconstructed using single-pixel detectors and far fewer samples than required by the Nyquist limit, *albeit* while still requiring many photons per pixel^22^,^23^.

Even for our longer acquisition times, our images have a very small (<20) number of detected photons per pixel, and thus, even for a uniform transmittance region of the object, the difference between neighbouring pixels in our images show a large variation inherent in the Poissonian statistics of the shot noise. Therefore, although the signal-to-background ratio of our images is high, the signal-to-noise ratio is not. However, the noise contributions in our images are well-defined both in terms of the Poissonian characteristics of photon counting and a known rate of noise events.

Real images are usually sparse in the spatial frequency domain, meaning they contain comparatively few significant spatial frequency components, a concept that forms the basis of JPEG image compression. The concepts of compressed sensing allow us to utilize this sparsity to infer an image from fewer photons than necessary in standard imaging techniques. Here we modify the image data to maximize the sparsity of the contributing spatial frequencies while maintaining the likelihood of the resulting image within the bounds set by the Poissonian statistics of the original data.

We denote the measured number of photons for each of the *N* image pixels to be an integer *n*_*j*_ and the fractional intensity of each pixel of the modified image to be *I*_*j*_. Given an estimated dark-count rate of *ε* per pixel, the Poisson probability distribution of measuring *n* photons given a pixel intensity *I* is


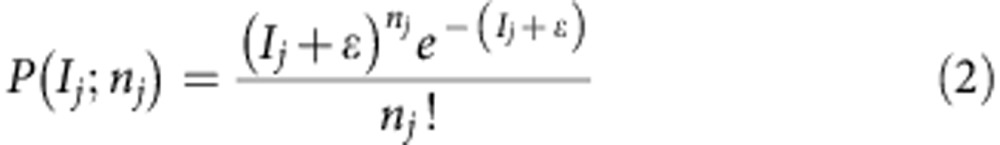


from which we can state the log likelihood of a modified image, *I*_*j*_, based on data *n*_*j*_ to be^24^


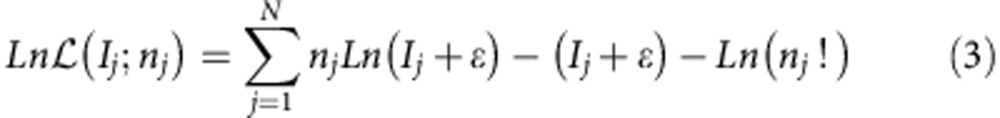


In the absence of any additional knowledge, the reconstructed image is simply the recorded data itself, that is, *I*_*j*_=*n*_*j*_. However, given that this data is subject to Poissonian noise it is reasonable to select an image from a large range of statistically plausible alternatives. Within this range, we choose to select the image, which has the sparsest discrete cosine transform (DCT). By defining the coefficients of the spatial frequencies of the whole image as *a*_*i*_, we can define a measure of sparsity through the number of participating spatial frequencies, *DCT_p_*, as


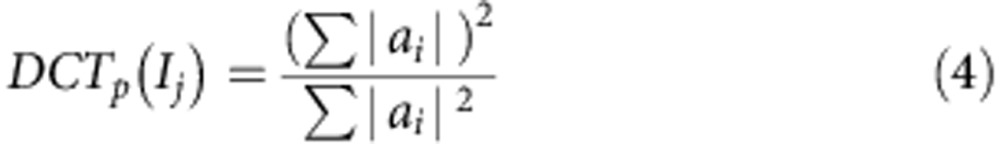


In our work, this optimization for *I*_*j*_ is based on an iterative maximization of a merit function, 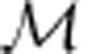, which combines the log likelihood of the reconstructed image and the participation function of its spatial frequencies as


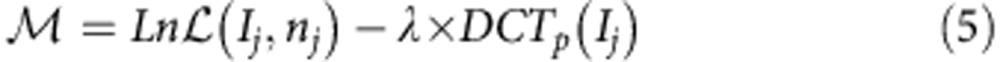


*λ* is a regularzation factor that sets the balance between a solution that satisfies the recorded data and a solution that satisfies the sparsity condition. Each iteration of our optimization routine makes a random change to the intensity value, *I*_*j*_, of a pixel selected at random. The merit function is calculated for this modified image, and repeated iterations are performed until the image corresponding to a maximization of this merit function is found. If *λ* is set to zero, the reconstructed image corresponds exactly to the data recorded, whereas if *λ* is set to a very high level, the reconstructed image corresponds to a uniform intensity distribution.

We use our imaging system in the GI configuration, as shown in Fig. 1b, where the object, the United States Air Force (USAF) test target, is placed in the heralding arm of the system and the photons detected by the heralding detector are used to trigger the ICCD camera. We acquire images based on the accumulation of an increasing number of frames and hence of an increasing number of photons and optimize each image using varying values of *λ*. Due to the point spread function of the intensifier in the ICCD, the observed resolution of the images is lower than the pixel size on the CCD. To better match the resolving power of our system to the pixel size in our reconstructed image, we spatially sum our image over adjacent pixels, such that the 600 × 600 pixels of the CCD are processed as a 300 × 300 image.

**Figure 3:**
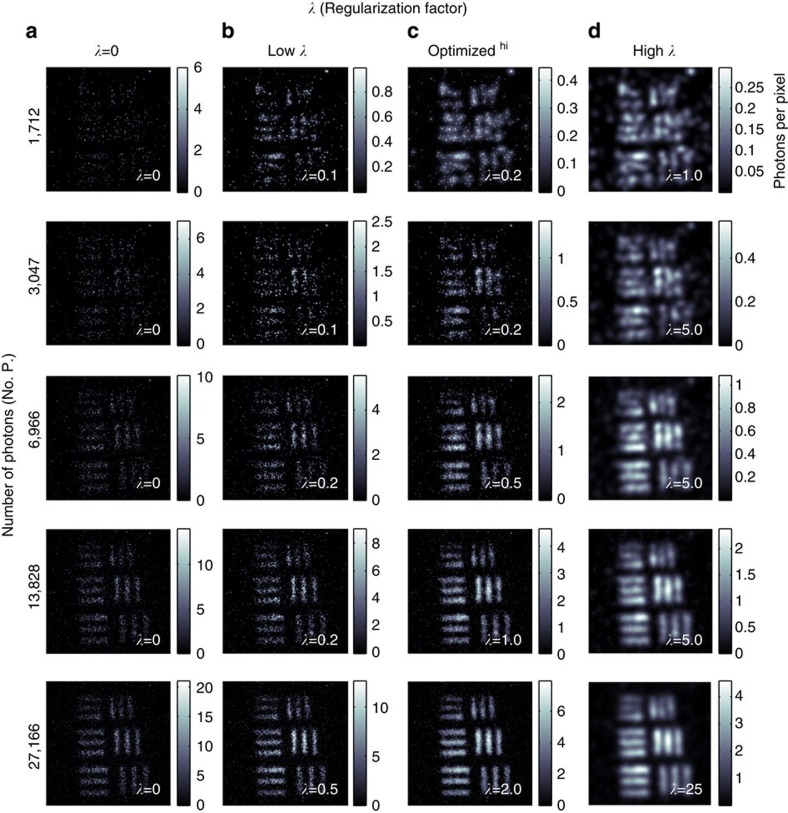
Regularized images of a USAF test target. Original data containing an increasing number of photons in the accumulated image in the left-hand column and reconstructed images for increasing values of *λ* in columns **b**–**d**. **b** shows the reconstructed images weighted towards maximizing the log likelihood, **d** shows the reconstructed images obtained when the optimization algorithm is overly weighted towards increasing the sparsity in the spatial frequency space and **c** shows the reconstructed images with *λ* adjusted to give subjectively the best images. Scale bar, 400 μm.

The reconstructed images, shown in Fig. 3, highlight the trade-off between changing the relative weighting between the log likelihood of the reconstructed image and participation of spatial frequencies within the merit function. As *λ* increases, the image becomes smoother due to increasing sparsity in the spatial frequency domain, but for high values of *λ* the resolution is degraded. Figure 3 shows the original data and images for a low-value, optimum-value and high-value of *λ*. The lower values of *λ* give images that retain the sparse characteristics of the original data, whereas the high values of *λ* give overly smooth images with associated loss of fine structure. The weighting factors for the central values of *λ* give subjectively the best images. We see that we are able to form a reconstructed image of the test target using an accumulated total of <7,000 photons, which corresponds to <0.2 photons per image pixel.

**Figure 4:**
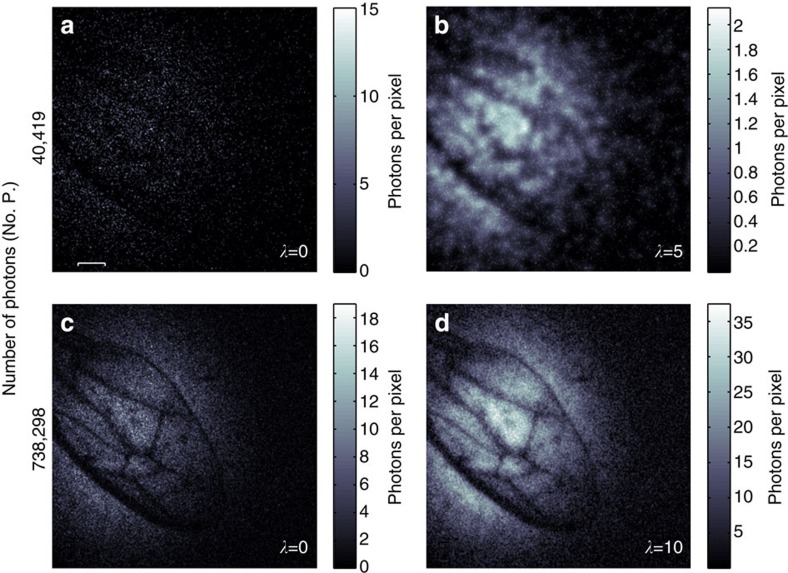
Regularized images of a wasp wing. How many photons does it take to form an image? (**a**) A weakly absorbing wasp wing imaged using 40,419 detected photons and (**b**) the corresponding reconstructed image. (**c**) An image of the same wasp wing with a greater number of photons and (**d**) its associated reconstructed image. Scale bar, 400 μm.

Having established that the system can be used in conjunction with a reconstruction technique to produce images from low numbers of photons, we apply the system to the imaging of a biological sample, in our case the wing of a household wasp. The data from this wasp wing for both low and high photon number acquisititions along with their reconstructed images are shown in Fig. 4. The low-photon number image comprises of only 40,419 detected photons over a field of view of 90,000 image pixels, corresponding to 0.45 photons per pixel.

## Discussion

For certain imaging applications a low-photon flux is essential, for instance in covert imaging and biological imaging, where a high photon flux would have detrimental effects. We have developed a low-light imaging technique using a camera-enabled, time-gated imaging system. We exploit the natural sparsity in the spatial frequency domain of typical images and the Poissonian nature of our acquired data to apply image enhancement techniques that subjectively improve the quality of our images. We show that it is possible to retrieve an image of a USAF test target using just 7,000 detected photons. These image enhancement techniques, combined with our photon counting, low-light imaging system, enable the reconstruction of images with a photon number less than one photon per pixel. As an example of low intensity imaging of biological samples, we use this time-gated ghost-imaging configuration to acquire low-photon number images of a wasp wing, with an average photon-per-pixel ratio of 0.45.

## Author contributions

M.J.P. conceived the experiment. P.A.M. and R.S.A. designed and performed the experiment. M.J.P., P.A.M., J.E.C.B. and R.S.A. designed the reconstruction algorithm. P.A.M., R.S.A., R.W.B. and M.J.P. analyzed the results. P.A.M., R.S.A., R.W.B. and M.J.P. contributed to writing the manuscript.

## Additional information

**How to cite this article**: Morris, P. A. *et al*. Imaging with a small number of photons. *Nat. Commun.* 6:5913 doi: 10.1038/ncomms6913 (2015).
